# Targeting protein phosphatases for the treatment of inflammation-related diseases: From signaling to therapy

**DOI:** 10.1038/s41392-022-01038-3

**Published:** 2022-06-04

**Authors:** Jie Pan, Lisha Zhou, Chenyang Zhang, Qiang Xu, Yang Sun

**Affiliations:** 1 State Key Laboratory of Pharmaceutical Biotechnology, Chemistry and Biomedicine Innovation Center (ChemBIC), Department of Biotechnology and Pharmaceutical Sciences, School of Life Science, Nanjing University, 163 Xianlin Avenue, Nanjing, 210023 China; 2 Jiangsu Key Laboratory of New Drug Research and Clinical Pharmacy, Xuzhou Medical University, 209 Tongshan Road, Xuzhou, 221004 Jiangsu China

**Keywords:** Inflammation, Immunological disorders, Inflammation, Target identification

## Abstract

Inflammation is the common pathological basis of autoimmune diseases, metabolic diseases, malignant tumors, and other major chronic diseases. Inflammation plays an important role in tissue homeostasis. On one hand, inflammation can sense changes in the tissue environment, induce imbalance of tissue homeostasis, and cause tissue damage. On the other hand, inflammation can also initiate tissue damage repair and maintain normal tissue function by resolving injury and restoring homeostasis. These opposing functions emphasize the significance of accurate regulation of inflammatory homeostasis to ameliorate inflammation-related diseases. Potential mechanisms involve protein phosphorylation modifications by kinases and phosphatases, which have a crucial role in inflammatory homeostasis. The mechanisms by which many kinases resolve inflammation have been well reviewed, whereas a systematic summary of the functions of protein phosphatases in regulating inflammatory homeostasis is lacking. The molecular knowledge of protein phosphatases, and especially the unique biochemical traits of each family member, will be of critical importance for developing drugs that target phosphatases. Here, we provide a comprehensive summary of the structure, the “double-edged sword” function, and the extensive signaling pathways of all protein phosphatases in inflammation-related diseases, as well as their potential inhibitors or activators that can be used in therapeutic interventions in preclinical or clinical trials. We provide an integrated perspective on the current understanding of all the protein phosphatases associated with inflammation-related diseases, with the aim of facilitating the development of drugs that target protein phosphatases for the treatment of inflammation-related diseases.

## Introduction

The concept of protein phosphorylation was proposed in 1955, arose from the determination of a dual requirement for adenosine triphosphate and “converting enzyme” (named as phosphorylase kinase lately) in vitro conversion of phosphorylase b to phosphorylase a.^1–3^ However, the enzyme converted the phosphorylase back to b called the “PR” enzyme (phosphorylase phosphatase) had been reported in the early 1940s.^4^ Phosphatases coordinate with protein kinases to control the homeostasis of protein phosphorylation modifications through their opposing activities. In many human diseases, the balance between protein phosphorylation/dephosphorylation is disrupted by the abnormal expression or unbalanced activities of these two enzyme types. In cancer, the aberrant activation of protein kinases is a feature of the pathological mechanisms; therefore, many oncogenic kinases have become targets in cancer therapies. Target-based drug discovery is the major strategy used in the pharmaceutical industry to identify new therapeutics. The current druggable targets are mostly ion channels, G protein-coupled receptors, and protein kinases, and many small molecule kinase inhibitors are already approved by the Food and Drug Administration for cancer therapy. By contrast, although protein phosphatases have been reported to regulate the process of inflammation in many inflammatory diseases through many signaling pathways in different immune or non-immune cell types,^5–11^ the functions of phosphatases and their inhibitors in human diseases are still unestablished.

**Fig. 1:**
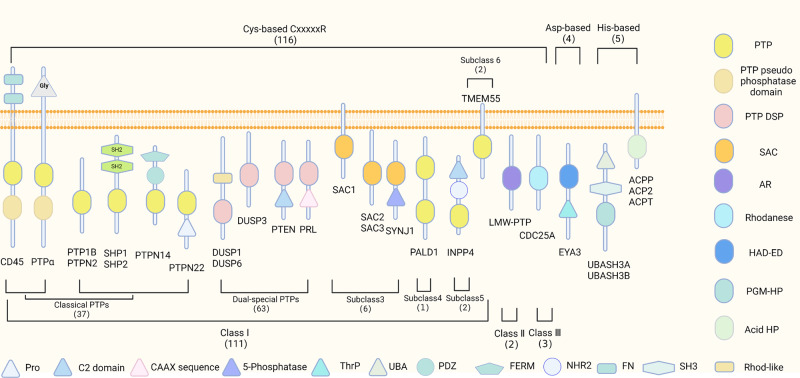
A growing tyrosine phosphatase family. Tyr phosphatases are classified based on their nucleophilic catalytic residue (Cys, Asp, or His). The representative schematic depiction and domain composition of the members in each group is shown. Cys-based PTPs contain the signature motif CxxxxxR with 3 classes and 6 subclasses. SAC phosphoinositide phosphatases and PTP-like phytases (PTPLPs) (PALD1/paladin), sharing the PTP fold, have been included in the class I Cys-based group as subclasses III and IV. INPP4 phosphatases (subclass V) and TMEM55 phosphatases have been included in the class I Cys-based group as subclasses V and VI. SSU72 has been included here as part of this class II category of PTPs. Class III phosphatases remain as previously classified.^13^ Non-Cys based Tyr phosphatases include the eyes absent (EYA) phosphatases recognized as Asp-based phosphatases. For His-based phosphatases, the ubiquitin-associated (UBA) and Src homology 3 (SH3) domain-containing protein (UBASH3) PGM phosphatases and the acid phosphatases (ACPs) have been incorporated into the extended PTPomes. PTP protein tyrosine phosphatase domain, DSP dual-specificity phosphatase domain, SAC Sac phosphatase domain, AR arsenate reductase, Rhodanese rhodanese phosphatase domain, HAD-ED HAD EYA domain, PGM-HP PGM-like HP domain, Acid HP His acid phosphatase domain, Rhod-like rhodanese-like domain, SH2 Src homology 2 domain, SH3 Src homology 3 domain, FN fibronectin type 3 domain, NHR2 Nervy homology 2 domain, FERM FERM (4.1/ezrin/radixin/moesin) domain, PDZ PDZ (PSD-95/Dlg/ZO-1) domain, UBA ubiquitin-associated domain, Thrp Thr phosphatase region; 5-Phosphatase, 5-phosphoinositide phosphatase domain, C2 domain C2 (C2A-Copine) lipid-binding domain, Pro proline-rich, Gly glycosylated

**Fig. 2:**
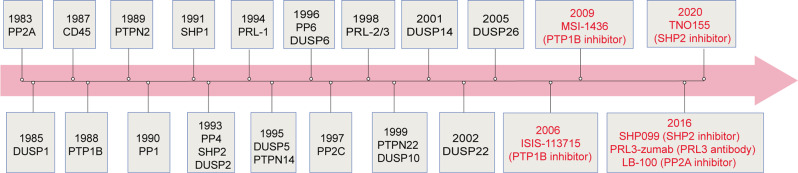
Timeline of the historical milestone for the discovery of protein phosphatases and their crucial inhibitors. SHP099 and TNO155 are SHP2 allosteric inhibitors. ISIS-113715 and MSI-1436 are PTP1B inhibitors. LB-100 is a PP2A inhibitor. PRL3-zumab is a monoclonal antibody of PRL3. The discovery of all the protein phosphatases take a long time and we show the time point for the first identified or cloned of all the protein phosphatases involved in the inflammation-related diseases (PP2A,^22^ DUSP1,^338^ CD45,^339^ PTP1B,^235^ PTPN2,^340^ PP1,^23^ SHP1,^341^ SHP2,^238^ DUSP2,^342^ PP4,^26^ PRL-1,^343^ PTPN14,^344^ DUSP5,^345^ DUSP6,^346^ PP6,^27^ PP2C,^17^ PRL-2/3,^347^ PTPN22,^348^ DUSP10,^349^ DUSP14,^350^ DUSP22,^351^ and DUSP26^352^) and the generation and development of their inhibitors in clinical trials (PRL3-zumb,^241^ LB-100^242^, ISIS113715,^236^ MSI-1436,^237^ and TNO155.^240^) SHP099 is the first allosteric inhibitor of SHP2 found by Novartis in 2016, which is a breakthrough in the study of allosteric inhibitor in the field of designing compound targeting protein phosphatases^239^

**Fig. 3:**
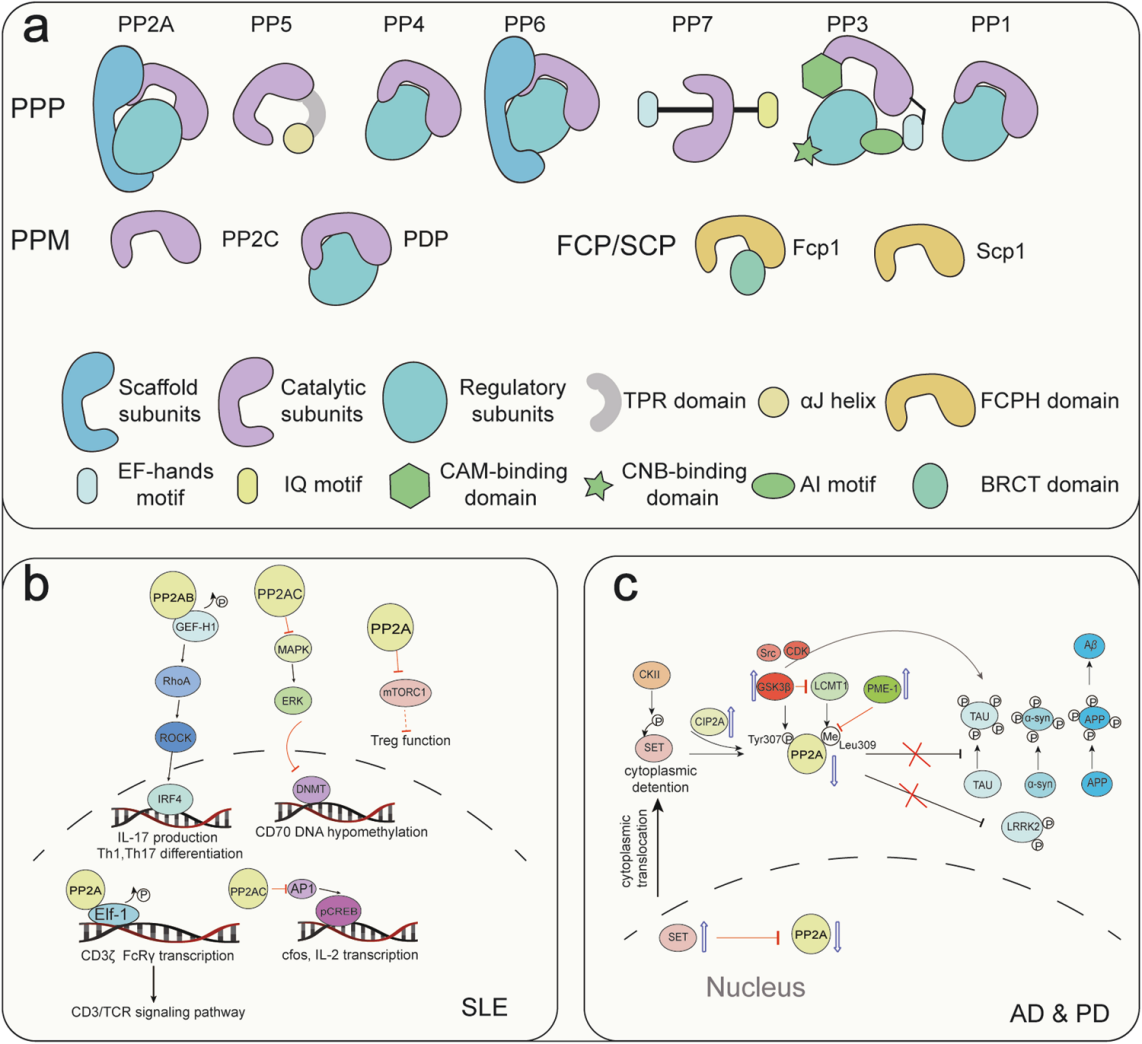
The PSP family and the regulated signaling pathways of PP2A in systemic lupus erythematosus (SLE), Alzheimer’s disease (AD), and Parkinson’s disease (PD). **a** Representative holoenzymes of 3 PSP families. The PPP family contains 7 subfamilies: PP1, PP2A, PP3, PP4, PP5, PP6, and PP7. The catalytic subunits are conserved. PP5 and PP7 work in monomeric enzyme form, while others work in holoenzyme form with the help of catalytic subunits, regulatory subunits, and/or scaffold subunits. The PPM family contains PP2C and PDP. PP2C works in monomeric enzyme form, and PDP works as heterodimer that consists of a regulatory and a catalytic subunit. FCP/SCPs have a conserved structural core of the FCPH domain. **b** The role of PP2A and its subunits in regulating immune cell differentiation and activation through major signaling pathways in SLE. The aberrant expression of different subunits of PP2A in SLE results in the activation of CD3 T cells, differentiation of Th1 and Th17 cells, and inhibition of Tregs. **c** The effects of PP2A on the accumulation of Tau, α-syn, and Aβ protein in AD and PD. In neurodegenerative conditions, nuclear pores start to leak, and SET is translocated to the cytoplasm. It then freely diffuses between the nucleus and cytoplasm, but phosphorylation of SET by CKII (casein kinase II) causes its retention in the cytoplasm. Together with an increased activity of GSK3β in the cytoplasm, the consequence is an increased tau phosphorylation, as indicated. PPP phosphoprotein phosphatases, PDP pyruvate dehydrogenase phosphatase, FCP TFIIF-associating component of RNA polymerase II CTD phosphatase, SCP small CTD phosphatase, APP amyloid-β precursor protein. TPR domain the tetratricopeptide repeat domain, FCPH domain FCP-homology domain, CAM-binding motif calmodulin binding motif, IQ motif calmodulin binding motif in PP7, CNB-binding motif calcineurin B binding motif, AI motif autoinhibitory motif, BRCT domain BRCA1 C-terminal domain like domain

The phosphatase types vary according to their amino acid substrates and include the Tyr phosphatases, such as src homology-2 domain-containing protein tyrosine phosphatase 2 (SHP2), protein tyrosine phosphatase 1B (PTP1B), and protein tyrosine phosphatase non-receptor type 22 (PTPN22), and the Ser/Thr phosphatases, such as type 2 A protein phosphatases (PP2A). Protein tyrosine phosphatases (PTPs) were first identified in the late 1980s, about 10 years after the first discovery of protein tyrosine kinases.^12^ Since then, studies using the conserved catalytic domain of PTPs to search the human genome database have identified and cloned 107 PTPs,^13^ which have extended to 125 PTPs currently^14^ (Figs. 1 and 2). The PTPs can be further divided into four diverse subtypes according to their structure and biofunction, as shown in Fig. 1. Class I PTPs are further divided into classic tyrosine-specific PTPs, and tyrosine and serine/threonine dual-specific phosphatases (DUSPs).^15^ The most recent study expanded the PTP superfamily to 125 members,^14^ whereas only about 30 protein Ser/Thr phosphatases (PSPs) have been identified. These PSPs are divided into three major families: phosphoprotein phosphatases (PPPs), metal-dependent protein phosphatases (PPMs), and the aspartate-based phosphatases (Fig. 3).^16^ PP2A is a representative member of the PPP family. The PPMs, including protein phosphatase 2 C (PP2C)^17^ and pyruvate dehydrogenase phosphatase, bind Mn^2+^/Mg^2+^ in their active centers.^18–21^ Aspartate-based phosphatases are represented by the TFIIF-associating component of RNA polymerase II CTD phosphatase (FCP) and the small CTD phosphatase (SCP). Initially, PP2A and PP1 were first discovered in 1983^22^ and 1990,^23^ respectively. In the next years, different subunits of PP2A were cloned continuously.^24,25^ Later, other PPPs and PPMs have been identified^17,26,27^ (Fig. 2).

## Inflammation and inflammation-related diseases

**Fig. 4:**
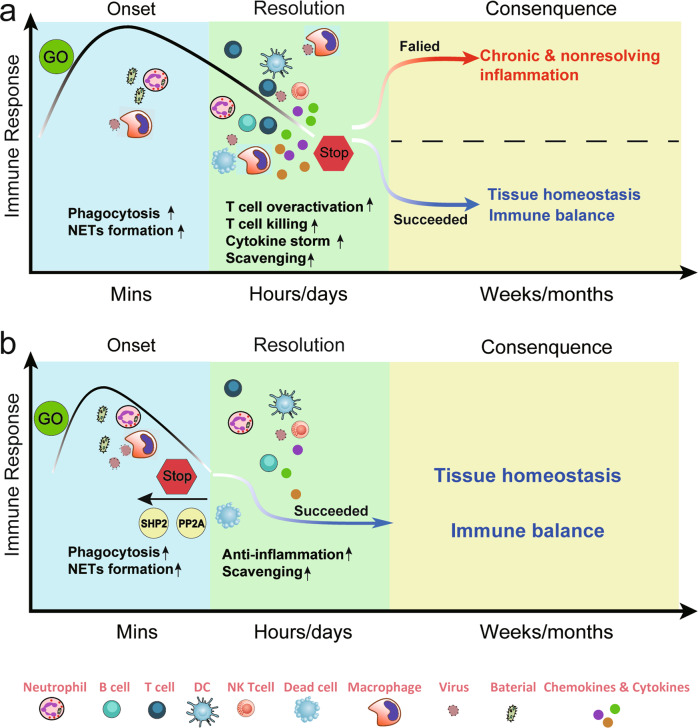
Immune response and the strategy of targeting phosphatases in inflammatory diseases. The immune response is a multiple process, and the detailed events differ over time. **a** At the onset of inflammation, innate immune cells are recruited to the injury or infection site within minutes and phagocytize bacteria. Subsequently, the adaptive immune cells infiltrate the site and secrete soluble cytokines, chemokines, or other cytotoxic proteins to activate T-cell killing functions and further scavenge debris. If the previous immune response succeeds in eliminating the infection, inflammation will terminate. If it fails, the proinflammatory immune response will continue for days, months, or even years and lead to chronic inflammation diseases. **b** Targeting phosphatases will help to advance the process of the inflammation resolution by modulating the function of immune cells, including but not limited to T cells, macrophages, neutrophils, and NK cells. The net result is restoration and maintenance of tissue homeostasis

Inflammation is generally considered a pathological process and is induced mainly by environmental exposure, infection, tissue injury, and autoimmunity (a whole-body and systematic inflammatory process characterized by excessive or abnormal activation of immune cells to recognize and attack self-cells). Inflammation is accompanied by the classical features of pain, heat, redness, swelling (e.g., tumors), and immune cell infiltration in the short term and may ultimately result in the loss of the core function of the tissue or system in the long term.^28–30^ Therefore, inflammation represents a protective response that tends to restore tissue homeostasis by initiating innate immunity and subsequent adaptive immunity. If successful, acute inflammation is resolved, restoring normal tissue architecture. If not, inflammation can then represent an irreparable deviation, and the inflammatory process can persist, develop into chronic and nonresolving inflammation, and finally cause tissue damage^31^ (Fig. 4a).

Inflammatory diseases have common physiological and pathological characteristics arising from excessive, aberrant, and continuous immune responses in immune cells and inflammatory responses in stromal cells.^32,33^ The dysregulation of immune responses causes chronic and continuous inflammation within certain tissues or systems and can lead to disability, economic burden, and psychological pressure. Thus, controlling the development of inflammation processes as early as possible is a promising strategy for the treatment of inflammatory diseases. However, the common nonsteroidal anti-inflammatory drugs and anti-cytokine therapies used in the treatment of inflammatory diseases rely on inhibiting the synthesis or action of mediators, such as IL-17 and TNF-α. Increased efforts are needed to develop drugs that can resolve inflammation. Notably, the aberrant expression, activation, or mutation of protein phosphatases are considered risk factors for autoimmune disease and are associated with an increased occurrence of many inflammatory disorders, mostly due to their involvement in the process of inflammation.^5,34^ Therefore, selective targeting of phosphatases should promote the resolution of inflammation processes (Fig. 4b).

The aim of this review is to provide a better understanding of the various functions of protein phosphatases and the signaling pathways that show promise for controlling the action of protein phosphatases in the pathogenesis of different inflammatory diseases. We also describe the prospective effect of phosphatase inhibitors for the control of the inflammation response in inflammatory diseases.

## The signaling pathways regulated by the protein phosphatases in inflammatory diseases

### SHP2 in inflammatory diseases

**Fig. 5:**
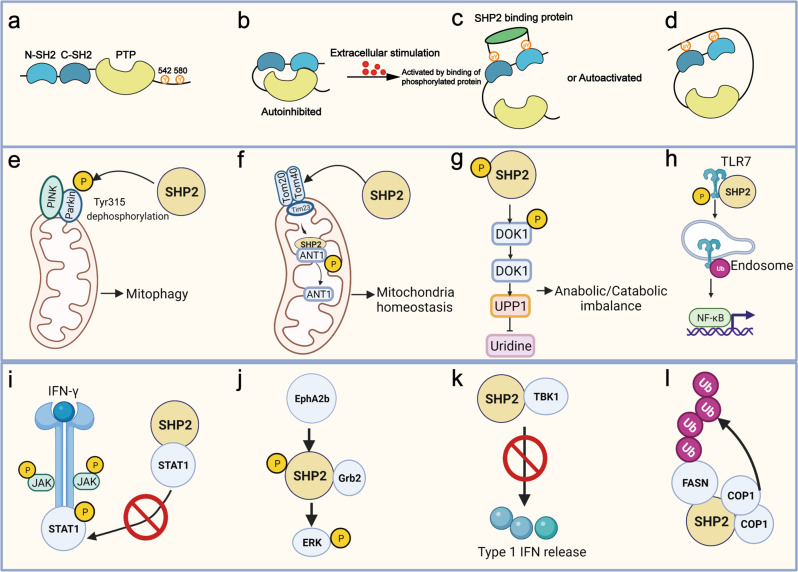
The structure of SHP2 and its function-dependent or independent of phosphatase activity. **a**–**d** A schematic diagram of the activation state of SHP2. **a** The core structure of SHP2. SHP2 consists of two SH2 domains at the N-terminal (N-SH2 and C-SH2), one PTP catalytic domain, and two phosphorylated tyrosine residues at the C-terminal. **b**, **c**, **d** When stimulated with extracellular signals, SHP2 either converts from an autoinhibited state to an activated state upon the binding of phosphoproteins to its SH2 domain or it is autoactivated through phosphorylation at its Tyr site. **e**–**h** Four representative phosphatase-dependent functions of SHP2. **e** SHP2 is translocated into mitochondria, dephosphorylated the Tyr315 site of parkin and further activated by mediated mitophagy.^38^ **f** In acute colitis conditions, SHP2 is moved into the matrix of mitochondria to dephosphorylate ANT1^112^ to maintain mitochondria homeostasis and inhibit inflammation responses. **g** In osteoarthritis, SHP2 dephosphorylates DOK1, which promotes UPP1-mediated uridine inhibition.^330^ **h** SHP2 dephosphorylates TLR7 and subsequently promotes TLR7 ubiquitination, which activates NF-κB. **i**–l Phosphatase-independent function of SHP2. **i** Phosphorylated SHP2 blocks the recruitment of STAT1 to IFN-γR during IFN-γ signaling, STAT1 homodimerization, and nuclear translocation.^46^ **j** EphA2b phosphorylates SHP2 and subsequently increases the binding of growth factor receptor-bound protein 2 (Grb2), which activates ERK signaling.^244^ **k** The C-terminal domain of SHP2 directly binds to the kinase domain of TBK1 to inhibit TBK1-mediated type I IFN signaling pathway.^332^ **l** SHP2 promotes the degradation of fatty acid synthase (FASN) by recruiting the binding of COP1, an E3 ligase^353^

SHP2, encoded by the protein tyrosine phosphatases non-receptor type 11 (*PTPN11*) gene, is a non-receptor PTP^15,35^ that is widely expressed in a variety of cell types in many organs and tissues. SHP2 consists of two SH2 domains at the N-terminal tail, a PTP catalytic domain, two tyrosine phosphorylation sites at the C-terminal tail (Tyr542 and Tyr580, which are responsible for the activation of SHP2), and a proline-rich region^36^ (Fig. 5). SHP2 is the best known target of PTPs and plays a key role in the transduction of many signaling pathways. It is responsible for the regulation of many cell events though its interaction with its substrates, both dependent and independent of its phosphatase activity (Fig. 5, Tables 1, 2).

**Table 1:** Interaction between SHP2 and its binding protein in inflammatory diseases

Protein	Function of SHP2	Regulated signaling pathway	References
ANT1	Dephosphorylation	SHP2-ANT1-NLRP3	^112^
TLR7	Dephosphorylation	SHP2-TLR7- NF-κB	^91^
DOK1	Dephosphorylation	SHP2-DOK1-Uridine	^330^
Parkin	Dephosphorylation	SHP2-Parkin- Mitophagy	^38^
PD-1	Dephosphorylation	SHP2-PD-1-M1 polarization	^113^
CagA	Activation	CagA-SHP2-IFNγ	^42^
p85	Dephosphorylation	SHP2-p85	^110^
STAT1	Dephosphorylation or sequestration	SHP2-STAT1-NF-κB	^46,148,331^
JAK2	Dephosphorylation	SHP2-JAK2-STAT3	^45^
RhoA	Dephosphorylation	SHP2-RhoA	^53^
D1R	Activation	DIR-SHP2- ERK1/2	^73^
cKit	Interactor	cKit-SHP2- AβPP	^76^
Tau	Interactor	Tau-SHP2- NGF Ras-MAPK	^77^
Gab1/2	Dephosphorylation	SHP2-FGF-Gab-MAPK	^61^
FGF	Dephosphorylation	SHP2-FGF-Gab-MAPK	^61^
EGFR	Dephosphorylation	EGFR-SHP2-Gab	^63^
CD31	Activation	CD31-SHP2-ZAP70	^97^
ZAP70	Activation	CD31-SHP2-ZAP70	^97^
TBK1	Adaptor	SHP2-TBK1-Type I IFN	^332^
Hook1	Interactor	SHP2-Hook1-TGF-β-EMT	^333,334^
Syntenin	Dephosphorylation	SHP2-sEV biogenesis	^41^
p115RhoGEF	Dephosphorylation	SHP2-DC migration	^335^
IL22R1	Dephosphorylation	SHP2-IL22R1-MAPK-STAT3	^336^
ASK1	Inhibits ubiquitination degradation	SHP2-SOX7-cJun	^337^
SIRPalpha	Being sequestered	IL4/IL13 and M2 activation	^47^

**Table 2:** The regulated signaling pathways of SHP2 involved in inflammatory diseases

Disease	Cell type	Signaling pathway	References
SLE	T cell	ERK-IFN-γ-IL-17A/F	^40^
Psoriasis	Macrophage	TLR7-NF-κB	^91^
	Neutrophil	ERK-NETosis	^93^
Systemic sclerosis	Fibroblast	TGF-β-JAK-STAT3	^55^
Contact dermatitis	T cell	AKT/STAT3	^95^
Osteoarthritis	Chondrocyte	DOK1-UPP1-Urdine	^100^
		Wnt/β-Catenin	^35^
		MAPK-NF-κB	^35^
Rheumatoid arthritis	Osteoblast	RUNX2/OSTERIX	^102,103^
	T cell	CD31-ZAP70.	^97^
	Fibroblast	IL17A-STAT3	^96^
Ankylosing spondylitis	Chondrocyte Osteoblast	BMP6/pSmad1/5	^104^
Parkinson’s disease	Neuronal cell	Parkin-Mitophagy	^38^
		FAK-LRRK2	^69^
		DIR-ERK	^71–73^
Alzheimer’s disease	Microglial cell	JAK/STAT	^70^
	Neuronal cell	cKit- A*β*	^76^
		Tau	^77^
Gastric inflammation	Epithelial cell	CagA-IFNγ/STAT1	^42^
		ERK/MAPK	^43^
		JAK/STAT-IL-6	^43^
IBD	Macrophage	IL-10/STAT3	^44^
		JAK2/STAT3	^45^
		SIRPα/CD47/SHP2/IL4/IL13	^47^
	T cell	STAT1-IFN-γ	^46^
Asthma	Fibroblast	IL6-STAT3	^55,56^
	Neutrophil	RhoA	^53^
Pulmonary fibrosis	Epithelial cell	TTF1-ABCA3	^61^
		FGF/GAB/ERK	^61^
	Fibroblast	STAT3-SOCS3	^56^
	Macrophage	IL4-JAK1-STAT 6	^60^
	Macrophage	M2 polarization	^60^
Lung inflammation		TGF-*β*-MMP12	^57^
	Epithelial cell	EGFR/Gab/MAPK	^63^
T1DM	T cell	mTOR/PD-1	^83^
	Macrophage	JAK-STAT-IFNI	^82^
T2DM	Macrophage	RAS-ERK-M2 polarization	^84^
		MAPK-endocytosis	^85^
		Akt/ERK	^86,87^
		IL-18	^88^
Non-alcoholic steatohepatitis	Hepatocyte	ROS-cJUN	^107^
Hepatic steatosis	Macrophage	ERK signaling	^88^

**Fig. 6:**
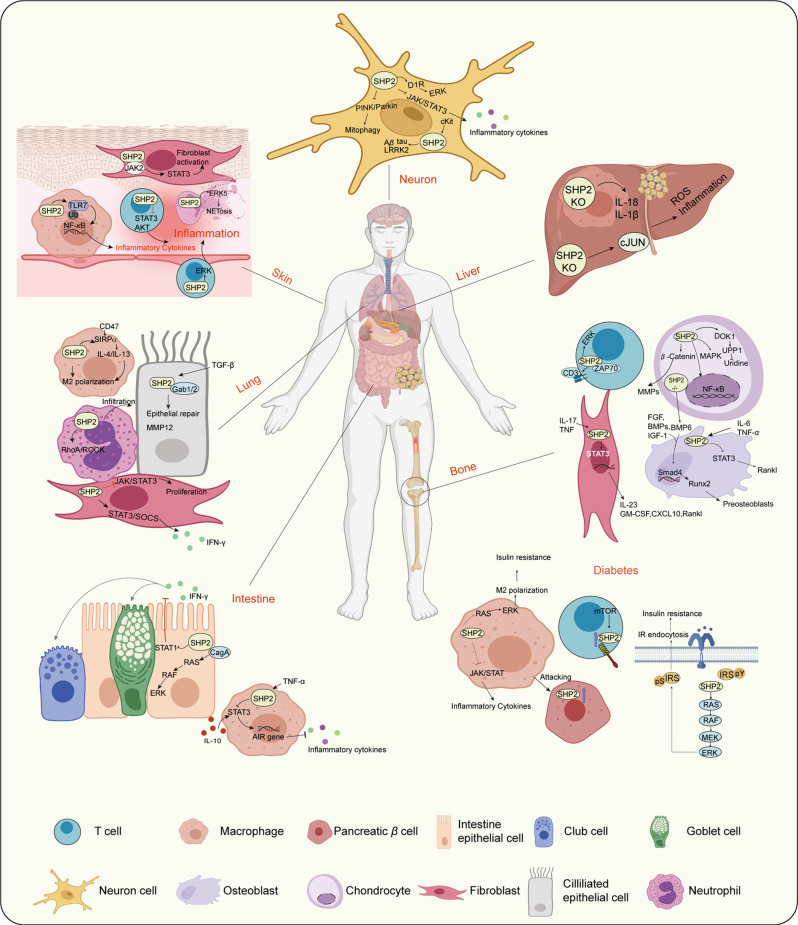
The regulating mechanisms of SHP2 in various inflammatory diseases. SHP2 plays a key role in the diverse inflammatory processes illustrated in the figure. SHP2 acts in many cellular signaling pathways in different cell types in inflammatory tissues. In neuroinflammation, SHP2 functions in neuron cells through involvements in the DIR/ERK, JAK/STAT3 and PINK/Parkin signaling pathways to regulate Aβ, tau, and LRKK2 accumulation, and inflammatory cytokine levels. In liver inflammation, SHP2 KO in liver resident macrophages or hepatocytes decreases liver inflammation by inhibiting IL-1β and IL-18 secretion and ROS production. In bone-related inflammatory disease, the different roles of SHP2 in T cells, osteoblasts, chondrocytes, and fibroblasts lead to bone inflammations, such as rheumatoid arthritis, ankylosing spondylitis, and osteoarthritis. In diabetes, abnormal expression of SHP2 in macrophages leads to insulin resistance and metaflammation and further attacks on pancreatic β cells. SHP2 in T cells interacts with PD-1 involved in T1DM. SHP2 also mediates RAS/ERK activation and insulin receptor endocytosis that lead to insulin resistance. In inflammatory intestinal diseases, SHP2 interacts with CagA, activates the RAS/RAF/ERK signaling pathway, and inhibits STAT1 in epithelial cells. SHP2 in macrophages also regulates IL-10 and proinflammatory cytokine secretion in the intestine, thereby affecting nearby club cells and goblet cells. In inflammatory lung diseases, SHP2 in lung epithelial cells, macrophages, neutrophils, and fibroblasts participates in lung inflammation by regulating the epithelial repair process, M2 polarization, neutrophil infiltration into lung tissue, and IFN-γ production. In skin inflammation, SHP2 activates fibroblasts, macrophage-mediated inflammation, T cells s and NETs formation that are responsible for skin inflammation. The figure is generated from BioRender.(https://app.biorender.com)

The abnormal expression of SHP2 or constitutive activation of its mutation promotes cancer cell proliferation and survival through RAS/MAPK signaling while also regulating tumor-infiltrating immune cells by serving as a downstream effector of the PD-1/PD-L1, TCR, CD28 pathway and the granulocyte-macrophage colony stimulating factor/ colony stimulating factor 1 receptor pathway in T cells and macrophages.^34^ SHP2 is a key regulator of immune cell-mediated inflammation through its direct influence on the immune response or the inflammation process.^30^ The functions of SHP2 in inflammatory diseases have been widely studied in inflammatory bowel disease (IBD),^37^ neuroinflammation,^38,39^ autoimmune diseases^40^ and lung inflammation^41^ as we next discussed (Fig. 6).

#### SHP2 in inflammatory gastrointestinal diseases

SHP2 activation is induced by binding to the cytotoxin-associated gene A (CagA) protein after *Helicobacter pylori* infection. This effectively causes interferon γ (IFN-γ) resistance and signal transducer and activator of transcription (STAT1) inactivation in human gastric cells, thereby promoting the progression of inflammation.^42^ The SHP2/ERK/MAPK and JAK/STAT signaling pathways are involved in immune cell infiltration into the gastric mucosa associated with IL-6 release during *Helicobacter pylori* infection.^43^ Inflammatory cytokines, such as TNF-α, have been shown to activate SHP2 in colon-resident macrophages and blood monocytes from IBD patients, and subsequently promote colon inflammation by disrupting the IL-10/STAT3 pathway.^44^ Activation of SHP2 in macrophages was also found to be essential in DSS‑induced colitis through dephosphorylation of JAK2/STAT3.^45^ SHP2 also interacted with cytosolic STAT1 to prevent the recruitment of STAT1 to IFN- γ and ultimately inhibited the release of proinflammatory Th1 cytokines.^46^ In wound healing response of intestine, SHP2 in macrophage was recruited and sequestered by signal regulatory protein α (SIRPα)/CD47 complex from IL-4 receptor or IL-13 receptor which promoted the IL-4/IL-13 signaling activation for wound healing.^47^ SHP2 can control the differentiation and function of secretory cells and goblet cells in the intestine to prevent inflammation.^48–50^ Mice that expressed the activated *Shp2*^*E76K*^ form specifically in intestinal epithelial cells showed sustained SHP2 activation and increased intestine and crypt lengths, which correlated with increased cell proliferation and migration.^50^ Collectively, SHP2 appears to serve dual roles in gastrointestinal inflammation, depending on its various functions in immune cells and stromal or epithelial cell types. It carries out its roles by variously regulating the ERK/MAPK, IL-10/STAT3, JAK2/STAT3, STAT1-IFNγ, JAK/STAT/IL-6, and CagA-IFNγ/STAT1 signaling pathways that mediate tissue damage and repair processes and the activation of inflammatory macrophages.

#### SHP2 in inflammatory lung diseases

Increasing evidence supports a role for SHP2 in inflammation-mediated pulmonary diseases, such as allergic asthma, pulmonary fibrosis, acute lung injury, and chronic obstructive pulmonary diseases.^51,52^ A high activity of SHP2 in pulmonary eosinophils of asthmatic children and in mice with allergic airway inflammation was associated with the dephosphorylation of p190-A Rho GTPase-activating protein.^53^ Similarly, fibroblasts from patients with severe eosinophilic asthma showed constitutive activation of SHP2, which resulted in negative regulation of IL-6-induced STAT3 phosphorylation. SHP2 activation in airway epithelial cells stimulated TGF- β1 production,^54^ which facilitated the conversion of fibroblasts to myofibroblasts and, in turn, induced collagen production^55,56^ and transforming growth factor β1 (TGF-β1)/MMP12-dependent emphysema.^57^ Conditional *Shp2* knockdown in airway epithelia in vivo and in vitro attenuated pulmonary inflammation.^54^ Notably, the inhibition or depletion of SHP2 had minor effects on the OVA-induced allergic reaction in mice.^58^ SHP2 was also shown to regulate the biogenesis of small extracellular vesicles by dephosphorylating its substrate syntenin Tyr46, while the knockdown of SHP2 promoted macrophage activation and lung inflammation.^41^ Increased expression of SHP2 in endothelial cells contributed to radiation-induced endothelial dysfunction and the subsequent establishment of an inflammatory microenvironment during radiation-induced lung injury.^59^ Therefore, most of the current evidence supports a proinflammatory role for SHP2 in asthma and lung inflammation.

In pulmonary fibrosis, SHP2 has prevented macrophage M2 polarization and the subsequent development of pulmonary fibrosis.^60^ Chang et al. demonstrated that SHP2 activation promoted hyporesponsiveness to IFN- γ in TGF-β differentiated myofibroblasts, and that TGF- β-activated SHP2 was tightly associated with pulmonary fibrosis.^56^ The protective role of SHP2 may be attributed to its inhibitory action on IL-4 mediated JAK1/STAT6 activation. SHP2 in alveoli epithelia has also been shown to mediate the expression of thyroid transcription factor 1 and ATP-binding cassette subfamily A member 3, which regulate surfactant protein expression during alveolar homeostasis. SHP2 also activated fibroblast growth factor (FGF)/ Grb2-associated binder family proteins (Gab)/ERK signaling, which is required for the lung epithelial repair program.^61^ Bleomycin-induced lung fibrosis tissues also showed a marked activation of SHP2, STAT3, and suppressor of cytokine signaling 3 that was highly associated with pulmonary parenchymal lesions and collagen deposition. Blocking STAT3 or SHP2 improved the antifibrotic efficacy of IFN-γ.^56^

SHP2 also activated NF-κB and macrophage polarization in the inflammation model during pulmonary infection.^62^ The results of cigarette smoke-induced chronic obstructive pulmonary disease models showed that exposure of mouse lungs or pulmonary epithelial cells to a cigarette smoke extract resulted in elevated levels of SHP2 with increased levels of IL-8, a proinflammatory cytokine, and lung inflammation via activation of Epidermal growth factor receptor (EGFR)/Gabs/MAPK.^63^ Therefore, although SHP2 mediates pulmonary diseases, its aberrant expression or activation plays a critical role in inflammatory pulmonary diseases.

#### SHP2 in neurodegenerative brain diseases

Neuroinflammation is recognized as a key process in the pathogenesis of neurodegenerative brain diseases, including Parkinson’s disease (PD) and Alzheimer’s disease (AD). Astrocytes, microglia, or other immune cells are responsible for the inflammatory response in the brain. SHP2 mediated the CXCL12α/CXCR4 signal responsible for guiding cerebellar granule cell migration.^64^ Aggregations of proteins, including prions, amyloid β, tau, and α-synuclein, as well as hypoxia and oxidative stress, lead to low-grade chronic inflammation in neuroglial cells.^65,66^ Liu et al. found a beneficial effect of SHP2 in alleviating PD in mice. In neuronal cells, SHP2 activated Parkin by dephosphorylation of Tyr315 and promoted the E3 ligase activity of Parkin, leading to SHP2-Parkin mediated mitophagy^38^ that protected against neuroinflammation.^67,68^ The enhanced kinase activity of the G2019S mutation of the *leucine-rich repeat kinase 2 (LRRK2)* gene has also been associated with familial PD.

SHP2 was shown to suppress neurite outgrowth by attenuating the activation of focal adhesion kinase (FAK), downstream of the *LRRK2* gene, through direct binding to and dephosphorylation of pTyr397-FAK.^69^ SHP2 also had an anti-inflammatory effect through negative regulation of JAK/STAT signaling, a proinflammatory signaling pathway in the brain.^70^ By contrast, SHP2 positively affected the development of PD in levodopa-induced dyskinesia. Several studies have reported that SHP2 interacts with dopamine receptor 1 and activates its target ERK signaling pathway during the progression of levodopa-induced dyskinesia.^71–73^ Hence, SHP2 has a dual role in PD, and its function depends on specific intracellular events.

Abnormal levels of amyloid-β and tau may initiate the production of the proinflammatory cytokines and chemokines that trigger the neuronal synaptic dysfunction and neuroinflammation associated with AD.^74,75^ SHP2 was shown to act downstream of cKit, which controlled the degradation of amyloid-β protein precursor. Treatment with SHP2 inhibitors or cKit inhibitors enhanced the phosphorylation of amyloid-β protein precursor, thereby reducing the accumulation of amyloid-β in neuronal cells.^76^ SHP2 also interacted with tau protein to form an SHP2-tau complex in neuronal cells. The formation of this complex was associated with the activation of SHP2 and the phosphorylation of tau.^77^ Overall, these results strongly indicate that SHP2 may be a positive mediator of amyloid-β and tau aggregation and neuroinflammation and therefore a promising therapeutic target.

#### SHP2 in diabetes

Type 1 diabetes mellitus (T1DM) is an autoimmune disease driven by the irregulated recognition of pancreatic β cells by an excess of active immune cells.^78,79^ By contrast, T2DM is a multifactorial metabolic disease associated with chronic inflammation.^80,81^ Yang et al. found that the elevation of SHP2 levels by *hsa_circ_0060450* circular RNA suppressed the JAK-STAT signaling pathway triggered by IFN-I to inhibit macrophage-mediated inflammation in T1DM.^82^ Another circular RNA, *hsa-miR-424-5p*, was reported to activate the mTOR signaling pathway and then to increase the expression of PD-1 in lymphocytes and upregulate SHP2, resulting in the pathogenesis associated with T1DM.^83^

The role of SHP2 in regulating type 2 diabetes mellitus (T2DM) has also been recently studied in patients suffering from Noonan syndrome and in a well-characterized mouse model that ubiquitously expresses the hyperactive mutation of *Shp2*^D61G/+^. Exploration of these model systems has revealed that systemic SHP2 hyperactivation promotes insulin resistance and constitutive inflammation of metabolic tissues. Hyperactivation of an SHP2 mutation directly promoted polarization of macrophages to the proinflammatory M2 phenotype. By contrast, depletion of macrophages or pharmacological inhibition of SHP2 improved insulin sensitivity and reduced metaflammation.^84^ Choi et al. similarly showed that insulin stimulation triggered a negative feedback pathway involving SHP2-MAPK that phosphorylated the insulin receptor substrate 1/2 and then promoted endocytosis, which inhibited the insulin receptor-mediated activation of PI3K-AKT signaling.^85^ SHP099, an allosteric inhibitor of SHP2, blocked this feedback regulation, prolonged insulin action on metabolism, and improved insulin sensitivity in a high-fat-diet-induced mouse model of diabetes.^85^ Two other studies also reported that SHP2 promoted insulin resistance by regulating the insulin receptor substrate through Akt/ERK signaling pathways.^86,87^ Liu et al. also demonstrated that conditional knockout of SHP2 in macrophages or pharmacological inhibition of SHP2 ameliorated high-fat diet induced hepatic steatosis and insulin resistance by elevating IL-18 levels.^88^ Therefore, SHP2 may be a strategic target for T2DM treatments.^89^

#### SHP2 in skin inflammation

A case report showed that a patient with Noonan syndrome-like symptoms and an SHP2 mutation showed an associated development of systemic lupus erythematosus (SLE), indicating the involvement of SHP2 in skin inflammation.^90^ Wang et al. also found increased activity of SHP2 in splenocytes from lupus-prone MRL/lpr mice or in peripheral blood mononuclear cells from patients with SLE. Mechanistically, SHP2 inhibition reduced the number of immature T cells, restored overactivated ERK/MAPK signaling, and decreased the production of IFN-γ and IL-17A/F in T cells, two cytokines involved in SLE.^40^

In our own research, we elucidated that the high expression of SHP2 in macrophages infiltrating into psoriatic skin promoted the dephosphorylation of toll-like receptor 7 (TLR7) at Tyr1024 and subsequently led to hyperubiquitinated TLR7 trafficking to endosomes and an induction of the excessive activation of NF-κB that consequently caused psoriasis.^91^ Both SHP099 and TK-453, another SHP2 allosteric inhibitor, prevented the progression to psoriasis.^92^ We also recently found that SHP2 in neutrophils aggravated psoriasis by promoting the formation of extracellular neutrophil traps and the subsequent cell death known as NETosis via the ERK5 pathway.^93^ SHP2 also mediated keratinocyte proliferation, migration, and differentiation through ERK activation in an IL-22-mediated psoriasis-like model.^94^ These results indicated that the function of SHP2 in psoriasis varied depending on the specific immune cell type.

In systemic sclerosis, SHP2 controlled TGF- β-induced STAT3 activation to promote fibroblast activation. Inhibition or depletion of SHP2 promoted the accumulation of JAK2 phosphorylated at Tyr570, reduced JAK2/STAT3 signaling, inhibited TGF- β-induced fibroblast activation, and ameliorated dermal fibrosis.^55^ The upregulated activity of SHP2 contributed to the immunosuppressive effect of activated T lymphocytes by inhibiting cell proliferation, AKT signaling, and STAT3 signaling in an allergic contact dermatitis mouse model.^95^ In conclusion, although SHP2 appears to exert mainly a proinflammatory role in several types of skin inflammation, some exceptions confirm that an increased activity of SHP2 may also have an immunosuppressive effect on skin inflammation.

#### SHP2 in bone-related inflammatory disease

Rheumatoid arthritis, ankylosing spondylitis, juvenile idiopathic arthritis, and psoriatic arthritis are all inflammatory bone diseases with different causes and pathogeneses. Upregulation of SHP2 was reported following IL-17A simulation, and this further activated STAT3 to induce IL-23, granulocyte-macrophage colony stimulating factor, and receptor activator of nuclear factor-κB ligand (RANKL) expression in rat models of rheumatoid arthritis.^96^ Analysis of synovial tissue biopsies from patients with rheumatoid arthritis showed that CD31 was excluded from the center of the T/B cell synapses. CD31 served as an inhibitory regulator of T-cell activation by activating and recruiting SHP2 to the surface of T cells, while activated SHP2 further dephosphorylated the ZAP70 protein.^97^ Similarly, a comprehensive screening found that increased SHP2 expression promoted the invasion and survival of fibroblast-like synoviocytes in rheumatoid arthritis, whereas SHP2 knockdown impaired the production of TNF-induced proinflammatory cytokines and growth factors.^98^ Abnormal CpGs methylation of the *Shp2* enhancer also triggered fibroblast-like synoviocyte aggressiveness and joint inflammation in mice.^99^

Liu et al. also recently found high activity of SHP2 in samples from patients with osteoarthritis, a chronic articular disease associated with bone inflammation. The increased activity of SHP2 reduced the phosphorylation of DOK1, its target protein, at Tyr397, and this was associated with UPP1-uridine production that ultimately facilitated the progression of osteoarthritis.^100^ Consistent with these findings, SHP2 overexpression activated the WNT/β-catenin signaling pathway to upregulate the downstream proteins involved in matrix degradation. SHP2 also positively regulated the activation of the MAPK and NF-κB signaling pathways in chondrocytes treated with IL-1β and promoted increased cytokine secretion.^35^ SHP2 also participated in the STAT3/STAT1 inactivation and induction of the p38 MAPK signaling pathway by calcium phosphate and monosodium urate in the regulation of osteoclastogenesis and development of osteoarthritis.^101^ SHP2 also modulated osteoblast differentiation and skeletal homeostasis by upregulating RUNX2/OSTERIX signaling and suppressing STAT3-mediated RANKL production by osteoblasts and osteocytes.^102,103^ A novel population of chondrocytes deficient in SHP2 was determined to impede the fusion of the epiphyseal plate and promote chondrogenesis in the joint cavity and enthesis by promoting new ectopic bone formation via BMP6/pSmad1/5 signaling in ankylosing spondylitis.^104^ Thus, SHP2 serves as a key molecule in rheumatoid arthritis, joint inflammation, and osteoarthritis. Therefore, SHP2 inhibition may be a potent therapeutic strategy for these inflammatory bone diseases.

#### SHP2 in liver inflammation

Hepatocellular carcinoma is closely associated with liver inflammation, as over 90% of all hepatocellular carcinomas arise from liver injury and chronic inflammation.^105^ Liu et al. reported that knockout of SHP2 in mouse macrophages led to high-fat-induced insulin resistance and hepatic steatosis in association with increased inflammatory IL-18 and IL-1β secretion.^88^ A study conducted in hepatocyte-specific SHP2 knockout mice showed that SHP2 depletion attenuated hepatocyte proliferation and liver regeneration after partial hepatectomy. However, the same mice also developed hepatic inflammation and necrosis, as well as hepatocellular adenomas.^106^ A similar report also demonstrated that dual depletion of SHP2 and PTEN in hepatocytes induced early non-alcoholic steatohepatitis and elevated ROS and inflammation in the hepatic microenvironment via activation of cJUN.^107^ A molecular analysis conducted on a 6-year-old girl with Noonan syndrome and autoimmune hepatitis type 1 revealed a heterozygous mutation c.923 A > G (Asn308Ser) in exon 8 of the *SHP2* gene, but no further associations or underlying mechanisms have been reported for SHP2 regarding the occurrence of autoimmune hepatitis.^108^ Another study reported autoimmune diseases in 14% of the patients affected by Noonan syndrome and Noonan-related syndromes.^109^ Above all, the loss of SHP2 expression in specific cell types or an active *SHP2* mutation both led to liver inflammation under specific pathological conditions.

#### SHP2 in other inflammatory disorders

SHP2 also has a function in sepsis-related endothelial inflammation, peritonitis, kidney injury, and pancreatitis. In sepsis-related endothelial inflammation, SHP2 activity was reduced by intracellular ROS production, and this was accompanied by increased adhesion molecule expression and endothelial activation through the activation of p38 MAPK and NF-κB. SHP2 was directly bound to MyD88 via Tyr257 in the SH2 binding motif of MyD88 and further dephosphorylated the binding site of p8.^110^ In insulin-induced endothelial inflammation, SHP2 expression was upregulated through the p38 MAPK signaling pathway and restricted the production of NO in an eNOS-independent and arginase-II-dependent manner.^111^ In experimental peritonitis models, activated SHP2 translocated to the mitochondria and inhibited ANT1, thereby suppressing NLRP3-mediated inflammation in bone marrow-derived macrophages.^112^ In peritonitis, SHP2 participated in PD-1 engagement and then induced M1 polarization and inflammatory cytokine production by dephosphorylating the PD-1 receptor or ligand.^113^ Increased activation of SHP2-mediated hemorrhage, cecal ligation, and puncture-induced inflammation in the kidney by activating ERK1/2-STAT3 signaling.^114^ Genetic knockout of SHP2 in myeloid cells decreased the macrophage infiltration and inflammatory factor production usually seen after unilateral ureter obstruction, thereby preventing renal fibrosis.^115^ SHP2 was also upregulated in the early stage of acute pancreatitis via MAPK activation, indicating a correlation between SHP2 expression and acute pancreatitis.^116^ Myocardial fibrosis was a risk factor of hypertrophic cardiomyopathy, a study found that *PTPN11* mutation in Noonan syndrome with multiple lentigines led to SHP2 binding to protein zero-related, activating IL-6/STAT3 signaling and caused fibrosis in mice fibroblast.^117^

**Fig. 7:**
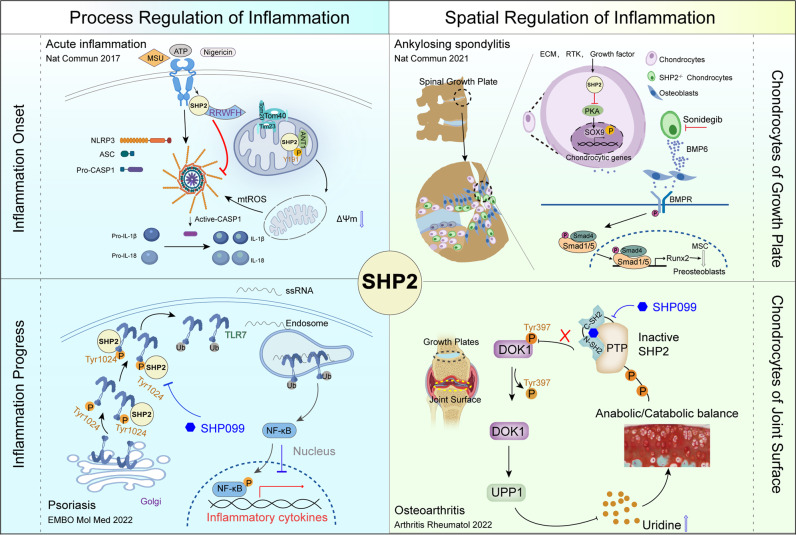
The process and spatial regulation of the inflammation response by SHP2. In the left panel, SHP2 responds quickly and translocates into the mitochondria to interact with ANT1 which inhibits the decrease in mitochondrial membrane potential and mtROS production at the inflammation onset. The early function of SHP2 in acute inflammation prevents the activation of NLRP3 and cytokine release. With the progression of inflammation, SHP2 promotes the TLR7/Endosome/NF-κB-mediated inflammation of psoriasis. Targeting SHP2 with SHP099 could relieve the progression of psoriasis. In the right panel, SHP2 is essential for the gene expression of chondrocytes of the growth plate. Decreased expression of SHP2 in growth plate chondrocytes results in overexpression of BMP6 and facilitates ankylosing spondylitis-like symptoms, while interruption with sonidegib prevents these symptoms. In the chondrocytes of the joint surface, an increased SHP2 expression disrupts the anabolic/catabolic balance and induces osteoarthritis by phosphorylation of its target protein DOK1 at Tyr397, which is associated with UPP1-uridine production. Targeting SHP2 with SHP099 may be a therapeutic strategy for osteoarthritis

In summary, SHP2 shows time- and space-dependent roles in inflammation by regulating several signaling pathways (Fig. 7). SHP2 may be a crucial molecular target for therapies aimed at resolving inflammation and treating inflammatory diseases. Therefore, strategies aimed at controlling SHP2 activity are pivotal. The most popular and advanced methods currently involve the use of SHP2 inhibitors, as discussed in the following sections.

### PP2A in inflammatory diseases

PP2A phosphatases are holoenzymes consisting of 3 subunits: a scaffolding subunit (PP2AA, α and a β isoforms), a catalytical subunit (PP2AC, α and a β isoforms), and a regulatory subunit (PP2AB). The PP2AA and PP2AC dimer can combine with diverse regulatory B subunits to form different PP2A heterotrimers (Fig. 3). The human PP2AB members are divided into four different families (B55, B56, PR48/PR72/PR130, and PR93/PR110).^118^ The different combinations of PP2AB subunits can yield, in theory, up to 100 different PP2A holoenzymes in human cells.^9,119^ Therefore, the function of PP2A is governed by how the three PP2A subunits combine. In addition, approximately one of three PP2A subunits can occur as a dimer consisting of just one A and one C subunit.^120,121^ PP2A is considered a tumor suppressor, and its activity and expression are decreased in many cancer cell.^122,123^ Restoration of PP2A activity through synergy with a MEK inhibitor, DUSP inhibitor, or CDK9 inhibitor is a promising strategy in cancer therapy.^122,123^

PP2A also has a role in inflammatory diseases, including neurodegenerative diseases (particularly AD) and autoimmune diseases.^10^ PP2A participates in inflammation processes through its regulation of the TLR/NF-κB, MAPK and tristetraprolin signaling pathways^10,124^ and through its role in T cells^125,126^ or other cell types. PP2A is the first Ser/Thr phosphatase recognized to contribute to SLE.^9^ The underlying mechanisms of PP2A in inflammatory diseases can be summarized as follows (Fig. 3b, c):

#### PP2A in SLE

Patients with SLE show increased expression of PP2A through both epigenetic and genetic regulation. The increased PP2A level regulates various signaling pathways that play essential roles in the pathogenesis of SLE.^9^ In SLE, PP2A was shown to dephosphorylate Elf-1 at Thr231 and to regulate the expression of CD3*ζ* and FcRγ in SLE.^127^ T cells have an FcRγ-phosphorylated spleen tyrosine kinase (pSyk) pathway that enhances the activity of the early CD3/TCR signaling pathway.^128^ Consistent with this, Syk positively regulated the expression of PP2A in T cells from patients with SLE.^129^

PP2AC has been identified as an SLE susceptibility gene that has an intronic SNP to control its transcription.^130^ Increases in PP2A promoted IL-17 production by increasing the activity of ROCK in T cells isolated from patients with SLE.^131^ Further study showed that the PP2A subunit PPP2R2A(PP2ABβ) directly interacted with guanine nucleotide exchange factor H1, leading to its dephosphorylation and activation in T cells and subsequent enhancement of the production of Ras homolog family member A (RhoA) and activated Rho associated kinases (ROCK).^132^ Genetic depletion of PPP2R2A (PP2ABβ) reduced Th1 and Th17 expansion, but not Treg differentiation, and reduced autoimmunity. By contrast, a requirement for PP2AC was demonstrated for the function of Tregs and the prevention of autoimmunity through suppression of the mTOR signaling pathway. Treg cell-specific depletion of PP2AC led to a severe and multi-organ autoimmune disorder that showed an SLE-like phenotype.^125^ The expression of PP2ABβ was deficient in SLE and led to resistance to T cell apoptosis induced by low IL-2 levels. The longer survival of autoreactive T cells contributed to the persistence of a longer immune response.^133^ The low level of IL-2 in SLE was attributed to increased PP2AC expression and inhibition of AP1 activity in SLE T cells through suppression of the binding of phosphorylated cAMP-response element binding protein (CREB) to the cfos and IL-2 promoter.^134^

The PP2AC subunit was overexpressed in SLE, resulting in the suppression of MAPK/ERK signaling pathway and promoted DNA hypomethylation of CD70, which was involved in the pathogenesis of SLE.^135^ The increase in PP2AC subunit expression upregulated the production of IL-17 in CD4^+^ T cells and resulted in more neutrophils in the peripheral blood, thereby promoting inflammation and facilitating the development of SLE.^127^ Targeting PP2A or its subunit in T cells may therefore be a potential strategy for the treatment of SLE.

#### PP2A in neurodegenerative diseases

Neurodegenerative diseases are considered a type of neuroinflammation. The role of PP2A in neurodevelopmental disorders has been well described, and abnormal phosphorylation of tau is observed in AD.^136^ PP2A has been identified as a tau phosphatase^137^ and is responsible for approximately 71% of the total tau phosphatase activity in the human brain.^138^ However, patients with AD show lower PP2A activity in both gray and white matter.^139^

PP2A has a leading role in AD, which has been well reviewed elsewhere.^140,141^ Tau is phosphorylated as a result of the crosstalk between protein kinases, such as glycogen synthase kinase-3β (GSK3β), CDK, and ERK, and phosphatases, including PP2A.^142–145^ Mechanistically, PP2A is inactive in the nucleus and active in the cytoplasm, thereby preventing the hyperphosphorylation of cytoplasmic tau. Under neurodegenerative conditions and during aging, nuclear pores become leaky, and the SE translocation (SET) protein, a PP2A inhibitor, is translocated to the cytoplasm. In addition, phosphorylation of the SET at Ser9 by casein kinase II causes its retention in the cytoplasm. Together with an increased activity of GSK3β in cytoplasm, the result is decreased activity of PP2A and increased tau phosphorylation, which then leads to tau accumulation and AD progression (Fig. 3c).

Similarly, the activity of PP2A is decreased in PD and regulates the phosphorylation of LRRK2, α-syn, amyloid precursor protein, and tau. This protein phosphorylation is associated with PD.^146^ In an experimental PD model, rotenone induced an increase in a calmodulin–Src complex in SK-N-SH cells, thereby activating Src kinase. The Src kinase, in turn, phosphorylated PP2A at Tyr307 and inhibited its activity, which led to the phosphorylation of α-syn.^147,148^ PP2A also had an effect on the transformation of the M1/M2 microglial population and influenced neuroinflammation and neuronal functions.^149^

#### PP2A in other inflammatory diseases

PP2A is a universally expressed phosphatase that is reported to participate in many other inflammatory diseases. PP2A was involved in the TLR-induced IRE1a activation in rheumatoid arthritis. PP2A reduced Sox9, CREB, and PP2A activity during chondrogenesis was involved in the downstream signaling of FGF1, TGF-β, and parathyroid hormone-related peptide in signaling pathways involved in chondrogenesis and chondrocyte differentiation.^150^ These data identified PP2A as a potential target in inflammatory joint diseases such as OA. Treatment with a PP2A activator targeted tristetraprolin inflammation and prevented bone erosion.^124^

PP2A also plays critical roles in lung inflammatory diseases. A recent study found that PP2A regulated M1 polarization of pulmonary macrophages and promoted lung inflammation in mice exposed to ambient particulate matter. These PP2A responses were mediated through the formation of a complex with mTOR/p70S6K/4E-BP1 and suppression of B56α, leading to enhanced phosphorylation of mTOR, p70S6K, and 4E-BP1.^151^ In asthma, MID1, a disruptor of mitsugumin 53-insulin receptor substrate-1, decreased PP2A activity through association with its catalytic PP2AC subunit and induced airway hyperreactivity and inflammation.^152^ PP2A regulated glucocorticoid receptor nuclear translocation and corticosteroid sensitivity, possibly by dephosphorylation of glucocorticoid receptor at Ser226 and dephosphorylation of upstream JNK1 in severe asthma.^153,154^ PP2A was shown to control mast cell degranulation through dephosphorylation of Thr567 in the Ezrin/Radixin/Moesin signaling pathway.^155,156^

A few studies have also reported an association of PP2A in asthma, but only limited details were available for the signaling pathway regulated by PP2A.^157,158^ In encephalomyelitis, PP2A was essential for the differentiation of Th17 cells through the SMADs/RoRγt pathway and NLRP3 activation in microglia.^159,160^ PP2A also mediated the dephosphorylation of Ser3 and Ser5 in the pyrin domain of NLRP3 during the priming phase of inflammasome activation in colitis.^161,162^ Some reports have also implicated PP2A in COPD.^163,164^ In the opposite, PP2A activation was also involved in the arctigenin-induced protective role of diabetic kidney disease through inhibiting NF-κB signaling pathway.^165^ These effects are all attributed to the inhibitory effects of PP2A on the mediators of inflammation.

The mechanisms underlying PP2A effects in SLE and neurodegenerative diseases have been widely studied, and PP2A is a promising target for the treatment of SLE and neurodegenerative diseases. However, PP2A is also involved in inflammatory joint diseases,^150,166^ asthma,^152,158^ encephalomyelitis,^159^ colitis^161,167^ but knowledge of its regulating pathways is limited. More studies are needed to establish its underlying molecular mechanisms.

### PTP1B in inflammatory diseases

**Fig. 8:**
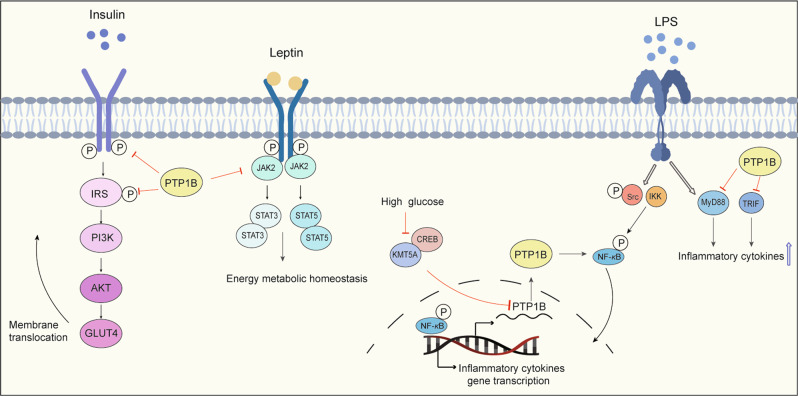
The regulated signaling pathways of PTP1B in inflammatory diseases. The role of PTP1B in insulin signaling, leptin signaling, and LPS-TLR-mediated inflammatory signaling in diabetes, obesity, and other inflammatory diseases. PTP1B inhibits the phosphorylation of the insulin receptor and insulin receptor substrate and their downstream PI3K/AKT/GLUT4 signaling, thereby preventing the translocation of GLUT4 to the membrane to transport glucose. PTP1B also inhibits JAK2 phosphorylation and attenuates the leptin JAK2/STAT signaling pathway to affect metabolic energy homeostasis. The CREB/KMT5A complex regulates PTP1B to modulate high glucose-induced endothelial inflammatory factor levels in diabetic nephropathy. PTP1B also acts as a negative regulator of TLR signaling via the suppression of both MyD88- and TRIF-dependent production of proinflammatory cytokines in macrophages stimulated by LPS

PTP1B, also known as PTPN1, consists of a C-terminal PTP domain, followed by tandem proline-rich regions near the C terminus. PTP1B is localized on the cytoplasmic face of the endoplasmic reticulum by means of a hydrophobic C-terminal sequence that imposes a topological constraint on the ability of PTP1B to access its substrates. PTP1B is widely reported as a key regulator of metabolic signals, including insulin signaling and leptin signaling, that negatively regulate the insulin pathway through JAK2-STAT3 and CREB/lysine methyltransferase 5 A (KMT5A), which are dysfunctional in T2DM and obesity^11,168,169^ (Fig. 8). Interestingly, a few studies have shown that PTP1B has a pro-neuroinflammation ability through its enhancement of the release of TNF-α, iNOS, and IL-6^170,171^ in the brain or GSK3β in PD.^172^ Recently, PTP1B was demonstrated to have dual roles in modulating the inflammation response in alcoholic liver injury^173^ and non-alcoholic fatty liver disease^174^ through its modulation of the inflammatory response. PTP1B was validated as a negative regulator of TLR signaling through its suppression of both MyD88- and TRIF-dependent production of proinflammatory cytokines and IFN-β in macrophages.^175^

PTP1B was found to have a role in other chronic inflammatory diseases. PTP1B deficiency ameliorated murine experimental colitis by activating the STAT3-JAK2 signaling pathway to promote the expansion of myeloid-derived suppressor cells.^176^ PTP1B also functioned as a critical negative regulator to limit allergic inflammatory responses.^177^ These reports indicated that PTP1B is a potential target for interventions in inflammatory diseases.

### Signaling pathways regulated by other phosphatases in inflammatory diseases

As summarized in the previous sections, SHP2, PP2A, and PTP1B are recognized therapeutic targets in many inflammatory diseases. However, other PTPs are also involved in the regulation of lymphocyte activation and autoimmunity.^178^ The most recognized receptor and non-receptor PTPs studied in inflammatory diseases include CD45, SHP1, PTPN2, PTPN14, PTPN22, and some members of the DUSP family.

Unlike the well-known SHP2, CD45 is a transmembrane protein that works as a receptor phosphatase. CD45 was critical in regulating the earliest steps in T-cell-receptor signaling and has a function in T cell activation and IL-2 production.^179^ In autoinflammatory osteomyelitis, CD45 promoted the onset and severity of IL-1β mediated bone inflammation by activating Src-family kinases but had no influence on ROS production.^180^ The absence of CD45 promoted hyperphosphorylation of Src-family kinases on their inhibitory tyrosine, thereby reducing their activity, immunoreceptor tyrosine-based activation motif signaling, and pro-IL-1β production, and ultimately resulting in disease alleviation.

SHP1 shares a high homology with SHP2 and has been reported to play essential roles in immune and inflammatory signaling pathways.^181^ It is also involved in neutrophilic dermatosis,^182^ rheumatoid arthritis,^183^ allergic inflammation, and anaphylaxis,^184,185^ as well as TNFα-induced endothelial inflammation,^186^ through regulation of IL-1 α/IL-1 αR signaling, Src and Syk kinases, and MyD88 signaling in different immune cells and/or epithelial cell and stromal cells.^187,188^

PTPN2, also known as T cell protein tyrosine phosphatase (TCPTP), dephosphorylates the insulin receptor, EGFR, Src family kinases, JAK, and STAT, and has a key role in IBD.^189,190^ PTPN2 regulates normal interactions between innate and adaptive immune cells and intestinal epithelial cells and has a protective function for the intestinal barrier.^191,192^ PTPN2 knockout or loss-of-function *PTPN2* SNP rs189321737 mutation led to macrophage polarization to the proinflammatory M1 phenotype through the regulation of IL-6 and STAT1. This limited autophagosome formation in response to invading pathogens and increased the permeability of the intestinal epithelial barrier.^193^ PTPN2 was also reported to suppress STAT‐induced inflammation in early diabetic nephropathy. This decreased periodontal inflammation in T2DM through dephosphorylation of colony‐stimulating factor 1 receptor at the Tyr807 site and other protein phosphatase substrates in the JAK1/STAT3 signaling pathway.^190,194^ PTPN2 blocked the process of macrophage inflammation by mediating p65/p38/STAT3 dephosphorylation in atherosclerosis.^195^ Overall, PTPN2 is considered an anti-inflammatory protein phosphatase that negatively moderates many inflammatory diseases.

PTPN14 has a known involvement in cancer, but its function in inflammation-related diseases is less clear. One study found that PTPN14 mediated the dephosphorylation and restoration of vascular endothelial cadherin at adherens junctions in LPS-induced acute lung injury, indicating a role for PTPN14 in inflammation responses.^196^ In a mouse model of LPS- and D-GalN-induced acute liver failure, PTPN14 initiated a cytokine storm by promoting ubiquitination of a suppressor of cytokine signaling and its downstream NF-κB signaling.^197^ In summary, the limited evidence in inflammation-related diseases indicates that PTPN14 may have an important role in the immune response and other inflammatory diseases.

PTPN22, also called lymphoid phosphatase, is mostly expressed in myeloid and lymphoid immune cells, and is rarely present in non-hematopoietic cell types.^198^ PTPN22 is a multifunctional regulator of immune signaling and inflammatory diseases in many types of immune cells. The C1858T polymorphism of PTPN22 has a widely reported association with increasing susceptibility in many autoimmune diseases, including T1DM,^199,200^ rheumatoid arthritis,^201–203^ and SLE,^204,205^ and other inflammatory diseases, including IBD,^206,207^ through its effects in T cells, B cells, and myeloid cells.

DUSPs can be divided into 5 further subtypes: mitogen-activated protein kinase/dual-specificity phosphatases (MKPs), phosphatases of regenerating liver-1 (PRL) family, myotubularin, PTEN, and atypical DSP subclasses that dephosphorylate pThr and pTyr.^15^ In this review, we mainly focused on those members reported to act on inflammatory diseases. PRL3, PRL1, and PRL2 belong to the DUSP family. Increasing PRL3 phosphatase activity has been shown to lead to higher susceptibility to colitis-associated colorectal cancer following inflammatory or mutational events.^208^ In MKPs, DUSP1/6 inactivates MAPKs by dephosphorylation of Thr or Tyr residues within the activation loop, thereby regulating many cellular signals in immune responses. DUSP1/6 were reported to serve as crucial regulators in PM2.5 exposure and COVID-19 infection or to induce proinflammatory immune responses in lung epithelial or endothelial cells through regulation of MAPK signals and NF-κB signaling.^209–212^ Vattakuzhi et al. reported that DUSP1 attenuated MAPK signaling, whereas its deficiency enhanced inflammatory osteolysis, which may be associated with osteolytic destruction.^213^ DUSP1/6 were both reported to have a function in cardiovascular diseases through inactivation of MAPK signaling.^214^ DUSP6 was shown to regulate intestinal inflammation by targeting the NF-κB or Nrf2 axis in macrophages.^215,216^

DUSP2 is localized in the nucleus and is predominantly expressed in hematopoietic tissues with high T-cell content. It regulates MAPK activation through the dephosphorylation of ERK/JNK/p38 following immune cell stimulation by extracellular stress or growth factors. Therefore, DUSP2 is involved in immune regulation and inflammatory diseases by, for example, enhancing the development of rheumatoid arthritis synovium and colitis and negatively regulating IBD.^217,218^

DUSP5 was reported to inhibit interleukin-1β -induced chondrocyte inflammation and to ameliorate osteoarthritis by inhibiting the NF-κB and ERK signaling pathways in rats.^219^ Analogously, DUSP5 mediated the protective effects against a high glucose‑induced inflammatory response by reducing the production of ROS and inflammatory cytokines.^220^

A recent review summarized the activity of DUSP10 in immunity and inflammation in different diseases.^221^ DUSP10 negatively regulated p38 and JNK activation induced by TNF-α in chondrocytes and exerted anti-inflammatory effects in osteoarthritis. Consistent with these findings, decreases in DUSP10 led to an inflammatory response in airway epithelial cells and aging diabetic mesangial cells through the activation of JNK and/or p38.

The atypical DSP subclasses have been the focus of only a limited number of studies in terms of inflammatory diseases. Que et al. demonstrated an effective neuroprotective effect of DUSP14 against an isoflurane-induced inflammatory response, pyroptosis, and cognitive impairment in aged rats through inhibition of NLRP3 activation.^222^ The expression of DUSP22 was significantly downregulated in T cells from patients with SLE, suggesting a contribution of DUSP22 T cell hyperactivation and subsequently increased inflammatory cytokine secretion.^223^ DUSP26 bound to transforming growth factor-β-activated kinase 1(TAK1) and blocked the activation of TAK1/JNK axis to alleviate inflammatory responses, insulin resistance, and hepatic steatosis.^224^ Overall, the members of the large DUSP family are involved in modulating many inflammatory diseases through different mechanisms.

In the protein Ser/Thr phosphatase family, apart from studies on the well-known PP2A, a few studies have also reported a role for PP1, PP4, PP6, and PP2C in inflammatory diseases. PP1 is a ubiquitously expressed protein Ser/Thr phosphatase consisting of a single catalytic subunit and one of nearly 200 regulatory subunits. PP1 was reported to have a function in antiviral innate immunity through the ablation of IKKε-stimulated IRF7 phosphorylation, and it dramatically attenuated IRF7 transcriptional activity that mediated IFNα production in host immune responses.^225^ PP1 also promoted lung inflammation through TNFα signaling in SARS-CoV infections, whereas its inhibitory subunit Kepi protected against SARS-CoV pathogenesis.^226^

PP4 consists of the PP4 catalytic subunit (PP4c) in association with different regulatory subunits that determine the substrate specificity. Given its role in innate immunity, PP4 has been initially reported as an activator of NF-κB-dependent transcriptional responses through its dephosphorylation of the c-Rel subunit of the NF-κB transcription factor.^227^ In viral infection conditions, direct binding of PP4 to TANK binding kinase 1 (TBK1) was reported to cause dephosphorylation of TBK1 at Ser172 and inhibition of TBK1 activation. This then restrained IFN regulatory factor 3 activation, resulting in the suppressed production of type I IFN and IFN-stimulated genes. Thus, PP4 acted as a negative regulator of RNA virus-triggered innate immunity.^228^ In experiment colitis, PP4 was pivotal for the maintenance of gut immunity through its positive regulation of Treg development and function.^229^ PP6, closely related to PP2A and PP4, has a bimetallic catalytic center and a known involvement in NF-κB signaling regulation. PP6 dephosphorylated TAK1 at Thr187 to modulate TAK1-induced NF-κB activity.^230^ NF-κB pathway activation was prolonged in keratinocytes derived from keratinocyte-specific PP6c conditional knockout mice.^231^ A recent study reported that PP6 deficiency in keratinocytes led to psoriasis-like skin inflammation and promoted IL-6 production through endosomal self-RNA sensing by dendritic cells.^232^

Another member of the PPM family, the well-studied PP2C phosphatase, Wip1, is expressed in hematopoietic progenitors, stem cells, neutrophils, macrophages, and B and T lymphocytes in bone marrow and peripheral blood and is responsible for the differentiation and function of immune cells during immune responses and inflammation. Wip1 knockout mice exhibited a proinflammatory phenotype that included increased production of inflammation-promoting cytokines, such as TNF-α, IL-6, IL-12, and IL-17, in skin and intestines through regulation of downstream targets, including p53, ATM, p38MAPK kinase, NF-κB, and mTOR.^233^ The expression of Wip1, which functioned as a negative regulator of NF-κB activation, was increased by inflammatory signals to maintain cell function and reduce inflammation.^234^

These studies have shown an association between different phosphatases and inflammatory diseases. Phosphatases play critical roles in modulating the phosphorylation of many proteins that are key mediators in many signaling pathways and in immune responses.

## Progress in innovative pharmacological approaches that target phosphatases

Research on phosphatase inhibitors can be traced back to the late 1980s.^235^ Professor Nicholas Kester Tonks, discovered PTP1B.^235^ Subsequent studies revealed that PTP1B is a key negative regulator in the insulin signaling pathway and have a role in diabetes. However, many first designed compound targeting PTP1B failed. A few years later, in 2006, the PTP1B inhibitor ISIS-113715 went into clinical trial,^236^ while the same as a clinical trial of PTP1B inhibitor, MSI-1436,^237^ it was terminated in 2008. These unsuccessful outcomes of PTP1B indicated the challenges of designing approximate protein phosphatases. Since SHP2 was first cloned in 1993 by professor Feng,^238^ the efforts to find its function and inhibitor have never stopped. The initial strategy to develop phosphatase modulators were focused on targeting the catalytic active sites. However, due to the highly conserved character of catalytic sites on phosphatases and the negative charges are required to guarantee the tight binding, it is difficult to achieve ideal selectivity and bioavailability for phosphatase modulators. Thus, for a long time, phosphatases were regarded as a kind of “undruggable” targets of small molecules. Excitingly, the molecular glue, known as allosteric inhibitor, that keeps the protein phosphatases in its autoinhibited conformation called allosteric inhibition opens new window for targeting phosphatases. The first reported SHP2 allosteric inhibitor in 2016, SHP099, was a breakthrough in the finding of allosteric inhibitor.^239^ In 2020, the derivative of SHP099, TNO155, went into clinical trial for the treatment solid tumor.^240^ In addition to PTP1B and SHP2 inhibitors, there are few drugs targeting other phosphatases, and only a few drugs such as PRL3-zumab^241^ (anti-PRL3 antibody), and LB-100^242^ (PP2A inhibitor) have advanced to the clinical stage (Fig. 2, Table 3). Fortunately, there are many potential compounds and strategies in many preclinical studies were demonstrated to targeting protein phosphatases as our next discussed.

**Table 3:** Drugs targeting protein phosphatases involved in inflammation in clinical trials

Compound	Target	Conditions	Phase	Status	Combination Therapy	NCT Number
TNO155	SHP2	NSCLC Esophageal SCC, Head/Neck SCC, Melanoma	Phase 1	Recruiting	Nazartinib	NCT03114319
		NSCLC Esophageal SCC, Head/Neck SCC, Melanoma	Phase 1	Recruiting	Spartalizumab or Ribociclib	NCT04000529
		Gastrointestinal Stromal Tumors				
		Colorectal Cancer				
		Advanced solid tumors with KRAS^G12C^ mutation	Phase 1/2	Recruiting	JDQ443	NCT04699188
		Advanced Cancer, Metastatic Cancer, Malignant Neoplastic Disease	Phase 1/2	Recruiting	MRTX849 or Dabrafenib, LTT462	NCT04330664
		BRAF^V600E^ Colorectal Cancer	Phase 1	Recruiting	Trametinib,	NCT04294160
BBP-398	SHP2	Advanced solid tumor	Phase 1	Recruiting	None	NCT04528836
JAB-3068	SHP2	NSCLC,	Phase1/2	Recruiting	None	NCT03565003,
		Head and Neck Cancer, Metastatic Solid Tumors				NCT03518554
RMC-4630	SHP2	Pancreatic Cancer,	Phase	Recruiting	Sotorasib, LY3214996	NCT05054725
		Colorectal Cancer, NSCLC	1/1b/2	or Active	Cobimetinib	NCT03634982
		KRAS mutation related tumors			Osimertinib	NCT03989115
						NCT04916236
RLY-1971	SHP2	Solid Tumor	Phase 1	Recruiting	None	NCT04252339
JAB-3312	SHP2	NSCLC, Colorectal Cancer, Pancreatic Ductal Carcinoma, Esophageal SCC, Head and Neck SCC, Breast Cancer, Other Solid Tumors	Phase 1	Recruiting	None	NCT04045496 NCT04121286
SH3809	SHP2	Advanced solid tumor	Phase1	Recruiting	None	NCT04843033
ERAS-601	SHP2	Advanced or metastatic solid tumors	Phase1	Recruiting	MEK inhibitor	NCT04670679
Sodium stibogluconate	SHP2	Myelodysplastic Syndromes	Phase 1	Terminated	None	NCT01009502
		Advanced Cancer		Completed	Interferon alpha-2b	NCT00629200
		Solid Tumors				
MSI-1436C	PTP1B	Metastatic Breast Cancer	Phase1	Terminated	None	NCT02524951
MSI-1436	PTP1B	Diabetes Mellitus	Phase1	Terminated	None	NCT00806338
ISIS-113715	PTP1B	Type 2 Diabetes Mellitus	Phase 2	Terminated	None	NCT00330200
PRL3-zumab	PRL3	Advanced Solid Tumors	Phase1	Recruiting	None	NCT03191682
LB-100	PP2A	Solid tumor	Phase1	Completed	Docetaxel	NCT01837667
		Recurrent Glioblastoma	Phase2	Recruiting	None	NCT03027388

### SHP2 inhibitors

The activity of SHP2 can be regulated by its interaction with its binding proteins, such as PD-1 and growth factor receptor-bound protein 2, in a phosphorylation-independent activation that releases SHP2 from its autoinhibited conformation. The phosphorylation status of SHP2 itself is important for activation.^243^ The kinase ephrin type-A receptor 2 and platelet-derived growth factor receptor-β were reported to mediate the Tyr542 and Tyr580 phosphorylation that activates SHP2.^244,245^ This Tyr542 and Tyr580 phosphorylation of SHP2 can be induced by insulin,^111^ fibroblast growth factor, platelet-derived growth factor,^246^ and mood changes^247^ by some as yet undetermined kinases.

**Fig. 9:**
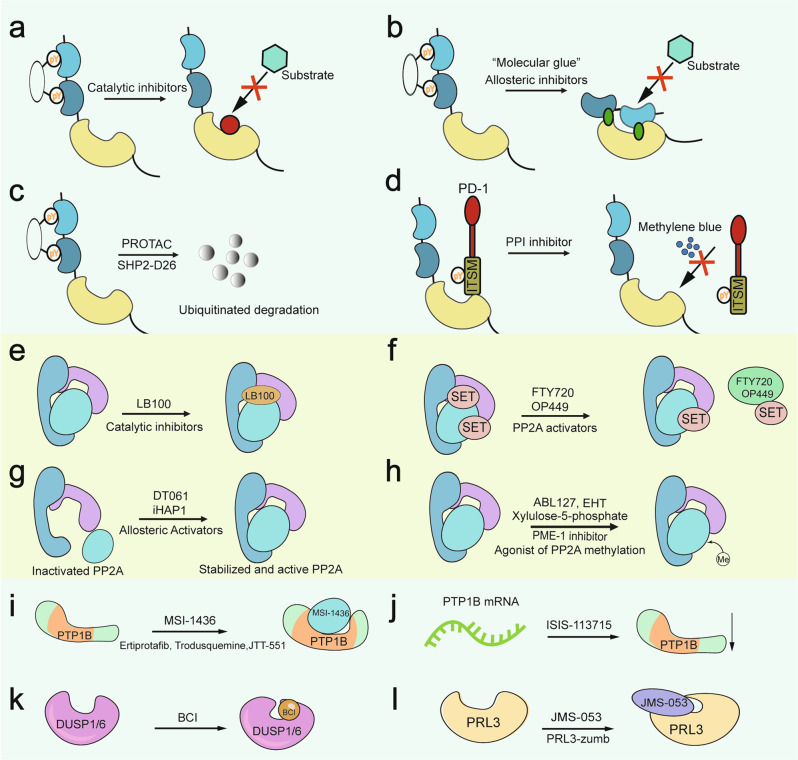
Modes of action of phosphatase inhibitors and activators. **a–d** The four known types of SHP2 inhibitors have diverse mechanisms that target the catalytic pocket of SHP2, that target the allosteric site of SHP2, that directly degrade the SHP2 protein, or that block the interaction between SHP2 and its substrates. **e–h** The inhibitor or activators of PP2A, including **e** a PP2A catalytic inhibitor undergoing clinical trials; **f** inhibitor of its endogenous inhibitory protein; **g** direct activator of PP2A; **h** post-translational modification of PP2A (e.g., methylation and phosphorylation). **i** Noncompetitive inhibitors of PTP1B bind to a site consisting of the last 20 residues of the catalytic domain. **j** The antisense oligonucleotide inhibitor of PTP1B messenger RNA reduces the translation of PTP1B protein. **k**, **l** JMS-053 and BCI are allosteric inhibitors of PRL3 and DUSP1/6, respectively

Several SHP2 inhibitors have been developed and are well discussed elsewhere;^36,248–254^ some of them have advanced to clinical trials.^253^ The most popular SHP2 inhibitors are catalytic activity inhibitors that target the PTP catalytic pocket, allosteric inhibitors that bind to a region outside the PTP catalytic pocket and maintain SHP2 in its autoinhibited conformation,^36,255^ and SHP2 Proteolysis Targeting Chimera (PROTAC) molecules^251^ (Fig. 9a–d). Small molecules that can interrupt the protein-protein interactions between SHP2 and its binding protein are also considered SHP2 inhibitors. However, strategies for targeting SHP2, apart from regulating its phosphorylation, remain limited.

The primary SHP2 inhibitor in use is the catalytic activity inhibitors, such as PHPS1,^77,100,216^ NSC87877,^250^ and II-B08^256^ that target the binding site of the PTP. Some natural compounds have been identified that inhibit SHP2.^257^ However, these inhibitors tend to have poor cell permeability or low oral bioactivity and lack the drug-like properties of successful drugs defined by the rule of five (RoF): logarithm of the octanol:water partition coefficient (log P) < 5, molecular weight <500 Da, number of H-bond donors (HBDs) <5, and number of H-bond acceptors (HBAs) <10.^258^ Thus, no SHP2 inhibitor targeting the PTP catalytic site has yet progressed to a clinical trial.

In 2016, SHP099 was introduced as the first identified allosteric inhibitor targeting SHP2.^239^ SHP099 was developed by scientists from NOVARTIS, and it represented a critical breakthrough in the development of SHP2 allosteric inhibitors.^259^ Currently, nine SHP2 inhibitors are undergoing clinical trials, and five of them (TNO155, JAB-3068, JAB-3312, RMC-4630, BBP-398, and RLY-1971) are allosteric inhibitors. Other allosteric inhibitors of SHP2 include SHP244, SHP389, SHP394, LY6, and RMC-4550.^36,253^ Notably, LY6 was much more selective at inhibiting leukemia cells carrying SHP2^E76K^ mutation than wild type cells.^260^ Therefore, LY6 may be a potential molecule for selective targeting of SHP2^E76K^ mutation-driven diseases. In general, the great efforts devoted to the development of the allosteric inhibitors of SHP2 indicate the great potential of these inhibitors in the clinical treatment of a variety of human diseases, including inflammatory diseases, in the near future.

Recently, a new method called PROTAC was introduced for the specific degradation of SHP2.^253^ The first PROTAC molecule discovered to target SHP2 was SHP2-D26.^261^ This molecule rapidly reduced SHP2 protein levels by more than 95% in KYSE520 esophageal cancer cells and MV411 acute myeloid leukemia cells. When compared to SHP099, SHP2-D26 showed a more than 30-fold higher activity for inhibiting the activation of ERK.^261^ A new report has shown that R1-5C, formed by conjugating RMC-4550 with pomalidomide using a PEG linker, acted as a potent and highly selective SHP2 PROTAC with high selectivity.^262^ Two other PROTACs targeting SHP2 by connecting pomalidomide with SHP099^251^ and by linking CRBN with an analogue of TNO155 using thalidomide^263^ were quickly designed thereafter.

Interfering with protein-protein interactions is another strategy that can inhibit the interaction of SHP2 with its substrates (Fig. 7d). Fan et al. found that methylene blue prevented SHP2 from binding with Y248-phosphorylated PD-1 and therefore enhanced the tumor-killing effect of CTLs.^264^ In addition, inducing the degradation and ubiquitination of SHP2 through its intracellular E3 ligase, FBXW7, was an outstanding approach that interrupted the downstream signaling pathways of SHP2, such as ERK and IFN I.^265^ Overall, although this approach is still in its infancy, it is a promising strategy for selective and effective inhibition of the effects of SHP2 (Fig. 7).

### PP2A activators and inhibitors

Pharmacological modulation mainly focuses on finding PP2A activators, but increasing the activity of an enzyme is typically complicated. Fortunately, the regulatory interactions of PP2A do provide opportunities for this. Three potential approaches are available for restoring PP2A activity: (1) inhibition of its endogenous inhibitory protein; (2) modulation of post-translation modifications of PP2A, such as methylation and phosphorylation; and (3) direct activation^266–268^(Fig. 9e-h).

PP2A undergoes post-translational modifications at specific amino acid residues. In particular, the Thr304-Pro-Asp-Tyr-Phe-Leu309 motif is modified by phosphorylation and methylation reactions that ultimately affect the activity of PP2A.^269^ Methylation of Leu309 increases PP2A activity, whereas phosphorylation of PP2A at Thr304 and Tyr307 decreases its activity. The phosphorylation of PP2A also alters its methylation, as Tyr307 phosphorylation inhibits the methylation of Leu309 by leucine carboxyl methyltransferase-1 and limits specific B subunit binding to the core enzyme. Similarly, phosphorylation of Tyr307 itself increases Tyr304 phosphorylation, although Tyr304 phosphorylation does not appear to influence methylation.^34,35^

GSK3β has also been shown to inhibit PP2A by upregulating the phosphorylation of the PP2A catalytic subunit and by further inhibiting the demethylation of PP2A at Leu309 through the upregulation of protein phosphatase methylesterase-1 (PME-1) and inhibition of leucine carboxyl methyltransferase 1 (LCMT1).^143,270^ CDK1 has been also identified as a kinase capable of phosphorylating Tyr304 of PP2A during mitosis^248,271^ The Tyr307 phosphorylation of PP2A is the most recognized marker of PP2A inactivation, but its regulating mechanisms are unclear. However, the kinases regulating PP2A phosphorylation have not been fully characterized.

Methylation of the Leu309 residue of the PP2A catalytic subunit is required for the binding of the regulatory B subunit^249,272^ The regulation of Leu309 methylation has therefore become an area of interest within PP2A targeted therapy. Protein phosphatase methylesterase-1 functioned to demethylate the Leu309 residue of the PP2AC subunit, which decreased the activity of PP2A.^273^ LCMT1 counters the activity of PME-1, which utilizes S-adenosylmethionine as a methyl group donor to transfer a methyl group onto the terminal carboxyl group of the L309 residue to regulate PP2A activity.^272^ Eicosanoyl-5-hydroxytryptamide has been shown to inhibit PME-1 and to facilitate the methylation of PP2A.^148^ However, no compound has yet been reported that can promote PP2A methylation by targeting LCMT1, although chloroethylnitrosourea and xylulose-5-phosphate have been reported to enhance PP2A methylation and increase its activity via other mechanisms.^274,275^

Within the cell, PP2A is inhibited by well-known cellular PP2A inhibitors that all have the ability to proactively inhibit active PP2A holoenzymes, including ANP32A, ANP32E, SET, CIP2A, ENSA, BOD1, ARPP19.^276^ These inhibitors either directly bind to the PP2A catalytic subunit or they target a very specific PP2A holoenzyme.^267^ Discovery of inhibitors of these PP2A inhibitory proteins, specifically SET and CIP2A, have provided insights into the mechanisms of PP2A dysregulation in many human diseases. Endogenous proteins or metabolites, including a phosphotyrosyl phosphatase activator^277–279^ and ceramide,^280–282^ have also been reported to activate PP2A. The phosphotyrosyl phosphatase activator binds to the PP2A dimer and may help to reactivate PME-1 binding to the catalytic subunit C of PP2A to promote the formation of PP2A homoenzymes. By contrast, ceramide activates PP2A by preventing the inhibitory protein SET from binding to PP2A. However, most studies on the regulation of PP2A activity are focused on its inhibitory proteins, such as SET and CIP2A, and only limited studies have explored how to promote PP2A activity through phosphotyrosyl phosphatase activator and ceramide. This may reflect the greater difficulty of improving protein expression or function compared to inhibiting a protein expression or function.

FTY720 (Fingolimod), a sphingosine analogue, was shown to activate PP2A by disrupting the interaction of SET to PP2A and consequently inactivating multiple PP2A-dependent substrates/pathways.^280,283^ Similarly, OP449 and ApoE mimetics also activated PP2A by binding to the C terminal of SET and disrupting the interaction between SET and PP2A.^284,285^ A small molecule, TGI1002, has also been found to disrupt the interaction between SET and PP2A and to increase PP2A activity.^286^

CIP2A inhibitors include bortezomib, erlotinib, and celastrol. Bortezomib was found to reduce the expression of CIP2 A at the transcriptional level, but with an unestablished mechanism.^245,246^ Decreases in CIP2A expression increased the activity of PP2A and led to AKT dephosphorylation at Ser473.^287–289^ Erlotinib and several of its derivatives have shown the ability to transcriptionally downregulate CIP2A expression by decreasing the binding of the Elk-1 transcript factor to the CIP2A promoter,^290–292^ but how the drug influences the Elk-1 is unclear.^293^ Celastrol binds to CIP2A directly and promotes its interaction with ubiquitin E3 ligase, CHIP, which is responsible for mediating the proteasomal degradation of CIP2A.^294,295^

Some small molecules have been identified that allosterically activate PP2A directly and are termed small molecule activators of PP2A (SMAPs).^296,297^ Phenothiazines and perphenazine were discovered to activate PP2A and dephosphorylate some of its targets, such as AKT.^298,299^ Iterative rounds of synthesis and optimization have since led to the development of several SMAPs.^296,300^

LB-100 is a small molecule inhibitor of the PP2AC subunit generated in 2016 and has recently completed a phase I clinical trial for the treatment of solid tumors in combination with docetaxel, although PP2A is widely accepted as a tumor suppressor.^301^ LB-100 inhibited homologous recombination repair in part by increasing the phosphorylation of the PP2A substrate CDC25C at the threonine 130 site. This resulted in activation of CDK1 and G2/M accumulation in many preclinical cancer models.^302,303^ Inhibition of PP2A with LB-100 has been reported to synergize with PD-1 to enhance the reactivity of microstatellite instability colon cancer to immunotherapy.^304^

Many other compounds are reported to activate PP2A, but the underlying mechanisms remain unknown. These compounds include carnosic acid,^305^ α-tocopheryl succinate,^306^ and forskolin.^307^ Overall, further studies should provide more insight into the mechanisms of targeted therapies for PP2A-driven human diseases, including inflammatory diseases.

### The druggable inhibitors of PTP1B and PRL3 and their advancement in clinical trials

The inhibitors of the PTPs involved in inflammatory diseases discussed above are shown in Fig. 8. Highly potent and selective PTP1B inhibitors have developed over the past 2–3 decades,^308^ and some are entering clinical trials.

MSI-1436 is a reversible and noncompetitive inhibitor of PTP1B that binds to the C-terminal regulatory region and another site consisting of the last 20 residues of the catalytic domain.^309^ However, the clinical trial of MSI-1436 was terminated at phase 1 for unknown reasons in 2009. Currently, some preclinical studies are ongoing for the PTP1B inhibitors ertiprotafib, trodusquemine, and JTT-551 for the treatment of diabetes.^310,311^ Another inhibitor being considered for treatment of type2 diabetes and obesity is ISIS-113715, an antisense inhibitor of the PTP1B gene. This molecule inhibits the translation of the PTP1B protein and is undergoing phase II clinical trials in 2006, however it terminated in 2009. The discovery of potent and specific PTP1B inhibitors is still vital for the treatment of diabetes and other PTP1B associated diseases.

JMS-053 and BCI are allosteric inhibitors of PRL3 and DUSP1/6, respectively.^312^ JMS-053 was found to bind to the site adjacent to the catalytic domain of PRL3 in 2016 and the other members, PRL1 and PRL2, to prevent the structural movement required for catalytic pocket function.^313^ The JMS-053 derivative NRT-870-59 shows a greater specificity than its parent JMS-053.^314^ However, the only drug specifically targeting PRL3 that has entered a clinical trial in 2016 for cancer treatment is a humanized antibody PRL3-zumab.^241,315^

BCI is an allosteric inhibitor that targets both DUSP1 and DUSP6. It binds to a pocket in the neighborhood of the catalytic sites of both DUSP1 and DUSP6 and thereby blocks the allosteric changes required for binding of the substrates.^316^ Unfortunately, although this inhibitor and others are used as tools to inhibit the activity of PTPs, such as PTP1B and DUSP, in research papers and even in clinical trials (Fig. 9 and Table 3), the discovery and innovation of inhibitors targeting PTPs has been far slower than for SHP2. New knowledge about their regulatory mechanisms and innovative drug development approaches are urgently required.

### Inhibitors of other phosphatases

Many phosphatase inhibitors have not advanced to clinical trials due to their low specificity, poor pharmaceutical properties, and weak bioavailability. For example, NSC-87877 is a small molecule competitive inhibitor of SHP2 that also shows inhibitory action against SHP1 and DUSP26.^317^ PTP1B inhibitors often lack selectivity over PTPN2 (TCPTP),^318^ but the catalytic activity of PTPN2 was recently shown to be auto-regulated by its intrinsically disordered tail and activated by integrin α-1. These findings may lead to a strategy for activating or inhibiting the activity of PTPN2 by targeting integrin alpha-1.^319^

The identification of catalytic-site-directed inhibitors of PTPs has been challenging due to the problem of creating cell membrane-permeable and highly negatively charged compounds. Therefore, phosphatase inhibitors targeting SHP1, PTPN2,^320–322^ PTPN22,^323^ and DUSPs^324^ are rare and limited to research or preclinical studies. Despite these various problems, the pace of research continues in the search for more druggable phosphatase inhibitors or other strategies, such as cell and gene therapy.

## Conclusions and perspectives

The various roles of phosphatases in many cell types in the regulation of downstream signaling pathways involved in inflammation make them promising drug targets for the treatment of inflammatory diseases. Moreover, the mechanisms of the protein phosphatases in inflammatory diseases or cancers are complex, which can be attributed to the diversity of its substrates. However, the current studies about protein phosphatases including the structure, function, and inhibitor are still limited compared to the kinases. The papers elucidating the newly found role and the regulated signaling pathways of SHP2, PP2A, and PTP1B in human diseases are springing up like mushrooms.

SHP2 is generally considered to accelerate tumor progression, a recent study showed that SHP2 deficiency also induced a tumorigenic and immunosuppressive environment in Myc-driven liver tumors.^325^ Thus, the use of activators or inhibitors of SHP2^326^ should be both taken into more consideration to provide precise therapeutic options. However, the study of SHP2 activators is limited to a few molecules or compounds, such as plasiatine,^327^ oleanic acid,^89^ trichomide A,^95^ fusaruside,^46^ and geranylnaringenin,^328^ and their mode of action requires further study.

PP2A is a widely accepted tumor suppressor^122^ and decreased activity of PP2A has been reported in association with SLE and AD. Most of the studied modulators of PP2A are PP2A activators. An exception is LB-100, a PP2A inhibitor that has been tested in tumor therapy in a clinical trial. A recent study also reported that PP2ACα increased in hepatic cells inhibited the expression of the hepatokine lecithin-cholesterol acyltransferase and the progression of hepatic osteodystrophy disease.^329^ Thus, targeting PP2A using LB-100 may be a viable strategy in this case.

PTP1B, as the early identified PTP, is an outstanding pharmacological target for obesity and T2DM. Numerous PTP1B substrates have been identified and some of which serve as key components in metabolic signaling including insulin, leptin, ER stress, cell-cell communication, energy balance. However, the development of PTP1B inhibitor is challenging and some novel approach such as targeting the interaction between the substrates and PTP1B may be easier and should be considered as a complementary approach.

In addition to the SHP2, PP2A and PTP1B, the function of other protein phosphatases in human diseases and the finding of their inhibitors are always on road^121^ and the pace of the study in the field of protein phosphatases never stops. The application of substrate-trapping mutant technology will help to define the physiological substrate specificity of members of the protein phosphatases. It will help to regulate the signaling pathways more precisely. Unlike the protein kinases, which function in a common manner, the protein phosphatases have evolved in separate families and some have several subunits that are structurally and mechanistically distinct. Protein phosphatases are a heterogeneous family (in subunit, expression, function, and regulation). Therefore, phosphatases are likely to be associated with many distinct pathologies in various human diseases. The phosphatases also have important normal biofunctions in non-diseased states. From the studies of our laboratory and others, the regulation of protein phosphatases for the control of inflammatory diseases depends on the timing of inflammation progression and the specific site at which inflammation occurs. Thus, a safer and more effective approach may be to disrupt the protein-protein interactions of protein phosphatase and its substrate or to target the phosphatase directionally at specific tissue sites and under specific inflammatory disease conditions to avoid the side effects of phosphatase modulators. To achieve this goal, optimization of the administration, drug design, drug delivery strategy, and other efforts will be needed. This could include the development of tissue-specific delivery of protein phosphatase inhibitors for the treatment of the special diseases, while not influencing other healthy organs.

Overall, knowledge of the crucial role of phosphatases in inflammation is progressing, but the drug discovery process remains focused on cancer therapy, with little emphasis on inflammatory diseases. More studies are urgently needed to establish the mutation, expression, or activity alterations of the protein phosphatases that influence the occurrence and progression of inflammatory diseases. Clinical studies are needed to determine whether these drugs are best used alone or in combination with current therapeutic agents. As further progress is made in defining the function of protein phosphatases and elucidating novel links to human disease, it is conceivable that new insights into therapeutic development will be revealed, either at the level of the protein phosphatases themselves or from targets within the pathways they regulate. We recommend that more attention should be given to phosphatase-targeted therapy for inflammatory diseases, and we hope that this will open up promising new avenues for developing effective drugs for inflammatory diseases through precise regulation of the inflammation process.
